# Chronic Myeloid Leukemia in Children and Adolescents: The Achilles Heel of Oncogenesis and Tyrosine Kinase Inhibitors

**DOI:** 10.3390/ijms22157806

**Published:** 2021-07-21

**Authors:** Maria Moschovi, Charikleia Kelaidi

**Affiliations:** 1 Division of Hematology-Oncology, First Department of Pediatrics, National and Kapodistrian University of Athens, “Aghia Sophia” Children’s Hospital, 11527 Athens, Greece; 2 Department of Pediatric Hematology and Oncology, “Aghia Sophia” Children’s Hospital, 11527 Athens, Greece; charikleia.kelaidi@gmail.com

## 1. Introduction

Chronic myeloid leukemia (CML) is a rare disease in children and adolescents. The goal of therapy in children and adolescents is normal life expectancy, without compromising normal growth and development and potential for achievement of milestones in adult life. The perspective of cure is also reflected in the goal of treatment-free remission, with its surrogate markers, such as deep molecular response, also becoming the new endpoints of therapy efficacy in children and adolescents. Chronic myeloid leukemia was a fatal disease to children and adolescents in the past. Following the treatment paradigm of imatinib, it became a chronic disease with the potential of complete remission and even cure without the long-term hazards of allogeneic hematopoietic cell transplantation. The diagnosis and treatment of CML affect a child’s trajectory through life and important physiological events like development and procreation.

**Figure 1:**
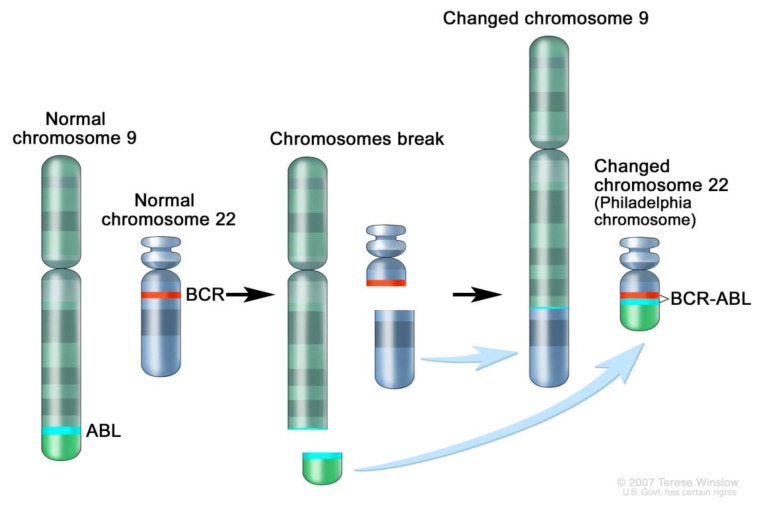
BCR-ABL fusion protein resulting from t(9;22) translocation. BCR-ABL is oncogenic by downstream activation of RAS, MEK and STAT pathways [1].

CML is a chronic myeloproliferative neoplasm that is characterized by the Philadelphia chromosome resulting from the t(9;22) translocation and its product, the BCR-ABL1 tyrosine kinase, with the protooncogene ABL1 being constitutively activated (Figure 1). Proteins P210, P190 and P230 derive from BCR-ABL depending on the breakpoint, with the former being the most frequent. Typically, the disease evolves through the chronic phase (CP), accelerated phase and blast crisis. In 2001, the introduction of the BCR-ABL1 tyrosine kinase inhibitor imatinib has essentially changed the natural course of the disease and produced considerable improvement in overall outcomes as compared with the available therapies at the time (interferon-α and hematopoietic stem cell transplantation). We will focus on CML mainly in children and adolescents and its chronic phase, while not dealing with the advanced phase or blastic crisis.

CML in childhood represents less than 10% of all cases of CML and 2–4% of all leukemias in children and adolescents with an incidence of around one per million [2,3]. CML occurs at all ages with an exponent of three in the incidence of CML per 5-year age classes, and may be diagnosed during infancy though, in general, it occurs after the age of 8 months. Most of the patients are ≥10 years old at diagnosis and boys are overrepresented (ratio 1.5). CML occurrence is a post-zygotic event (twin discordance). No transplacental transmission has been reported. While there is no evidence for CML-prone genetic syndromes, the disease may occur as a second malignancy or after autoimmune disease or radiation exposure.

## 2. Genomics

The BCR-ABL1 breakpoint may occur in either of two cluster regions, the major (M-BCR) and the minor (m-BCR) breakpoint cluster regions. The majority of children with CML carry the major breakpoint cluster region transcripts, similarly to adults. The transcript e13a2 (b2a2) is less frequent than e14a2 (b3a2) but its relative proportion is somewhat higher in children than in adults (39.6% in children vs. 37.9% in adults). The proportion of atypical, rare transcripts in children and adolescents is 3.25% in children aged 0–9 years and 1.87% in those aged 10–19 years. Breakpoint clusters occur in the vicinity of repeat regions more frequently in children as compared to adults, although the clinical significance of that variation is unknown.

Somatic mutations in cancer-related genes in addition to BCR-ABL1, detected at diagnosis of CML-CP, may independently affect overall prognosis and response to treatment. Interestingly, somatic ASXL1 mutations have been detected in six out of 21 children and young adults with CML-CP [2]. Variant allele frequencies were 4–46%. The frequency of somatic ASXL1 mutations was higher than that reported in unselected adults. That observation might prove clinically relevant and guide therapeutic choices for children in the future.

Before recognition of the Philadelphia chromosome as the cytogenetic hallmark of the disease and its widespread use for CML diagnosis, confusion between juvenile myelomonocytic leukemia (JMML) and pediatric CML was frequent in the literature and clinical practice [2]. However, as judiciously noted in an early report, JMML is neither chronic nor granulocytic, and CML was often referred to as chronic granulocytic leukemia. Before their clear distinction, classical Ph+ CML in children was named ‘adult-type CML’ as opposed to ‘juvenile-type CML’. The then acknowledged differences, still valid today, between the two diseases included colony-stimulating activity of the leukemic precursors and fetal erythropoiesis with increased HbF in JMML, and the presence of a Philadelphia chromosome in CML as opposed to monosomy 7 in JMML.

## 3. Treatment with Imatinib and the Tyrosine Kinase Inhibitors Era

**Figure 2:**
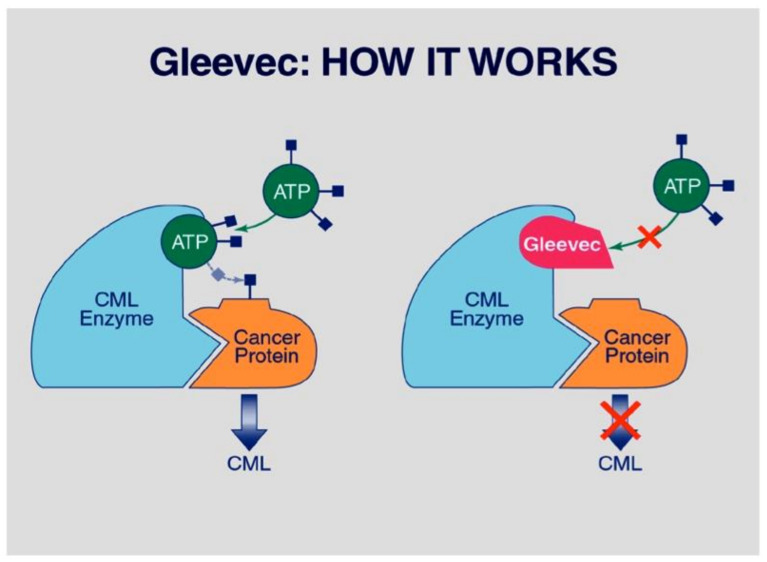
Mode of action of imatinib (Gleevec) in CML: Occupation of the tyrosine kinase pocket of ABL (CML enzyme) impedes use of ATP to phosphorylate its substrate and thereby inhibits oncogenesis. From Goodman, S.N.; Gerson, J. Mechanistic Evidence in Evidence-Based Medicine: A Conceptual Framework Internet. Rockville (MD): Agency for Healthcare Research and Quality (US); 2013 Jun. Results and Case Studies. Available online: https://www.ncbi.nlm.nih.gov/books/NBK154588 (accessed on 15 July 2021).

Imatinib was the first tyrosine kinase inhibitor (TKI) used in CML and one of the first targeted therapies used in medicine. By occupying the ATP-using kinase pocket of the BCR-ABL1 protein that is the Achilles heel of oncogenesis, imatinib blocks the tyrosine kinase in an inactive conformation and thereby inhibits its oncogenic activity (Figure 2). Inhibition of the tyrosine kinase activity results in a reduction in the leukemic burden. This mode of action, which earned TKI the designation of ‘targeted therapy’, differs from that of cytotoxic therapies, and explains why TKI treatment is lifelong, transforming CML to a chronic disease controlled by medication.

In 2003, the FDA approved imatinib for CML in children. Overall, results with imatinib in children compared favorably with those obtained in adults in late chronic or advanced phases of CM, with mild toxicity. Overall, in terms of overall survival (OS) and 5-year disease-free survival (DFS), mid-term results with imatinib were excellent. Moreover, a prospective comparison of children in CP receiving upfront imatinib versus allogeneic transplantation with a matched sibling donor (MSD) revealed superior long-term outcomes with imatinib. Accordingly, an epidemiological study comparing outcomes in the pre- and post-imatinib era showed dramatic improvements in outcomes [3]. Response evaluation timing and criteria are highly standardized from the European LeukemiaNet (ELN) recommendations for TKIs. Major molecular response (MMR), which corresponds to BCR-ABL1 transcript level of ≤0.1% on an international scale (IS), is the aim to be attained by 18 months of treatment. Deep molecular response includes MR4, 4.5 and 5, defined as BCR-ABL1 ≤0.01%, ≤0.0032% and ≤0.001%, respectively. In the phase IV clinical trial of imatinib in children with CP, 77% of the patients achieved complete cytogenetic response at a median time of 6 months (range, 6–20 months); 61% of the patients achieved complete cytogenetic response at the time point of 12 months.

### Prognosis and Side Effects

The application of established prognostic CML scores in children has generated inconsistent results. The EUTOS long-term survival score (ELTS) is a score established and validated in adults. Five-year DFS was 96%, 88% and 67% in patients with low, intermediate and high ELTS, respectively, while 5-year survival accounting for CML deaths was 99%, 96% and 89%, respectively.

Besides hematologic toxicity during the initial phase of treatment, the most frequent adverse event is musculoskeletal pain and cramps during the day and nighttime. Rare side effects reported in children include dermatological manifestations such as depigmentation or hyperpigmentation of the skin, mucosa and teeth and pseudoporphyria, and nephrological manifestations such as hypophosphatemia and nephrotic syndrome. The most preoccupying, well-documented, population-specific side effect of imatinib is the reduction in longitudinal growth rate with a direct effect of imatinib on the growth plate and bone mineral density in children. Initiation of imatinib at the prepubertal age is associated with more severe growth impairment. Regular endocrinologic evaluation +/− bone mineral density should be planned for prepubertal as well as pubertal patients. The Children’s Oncology CML Working Group recommends that a DEXA measurement of bone density be performed yearly, adequate calcium intake ensured and vitamin D maintained at adequate levels. Additionally, control of bone age and of levels of sex steroid hormones and gonadotropin is recommended in the case of growth or puberty delay, respectively. In order to mitigate the impact of imatinib on growth, especially in prepubertal children, but also to improve compliance, intermittent imatinib administration for three weeks a month (on/off schedule), was studied. Bone metabolism and growth rate improved under the intermittent schedule in patients suffering from late effects.

In the long term, one third of newly diagnosed children with CML-CP discontinued imatinib. Reasons for discontinuation were intolerance, resistance and lack of compliance [4,5,6]. Two second-generation TKIs, dasatinib and nilotinib, were developed as second line treatment in this setting and were approved in the frontline setting as well, also in children.

Dasatinib has higher potency and a broader spectrum of tyrosine kinase inhibition than imatinib. Dasatinib is also approved in children with newly diagnosed CML in CP and in children who are intolerant or resistant to imatinib. Rash and diarrhea are the more frequent dose-limiting toxicities. QTc must be monitored. Growth deceleration might occur. Nilotinib in children showed sustained responses. Prescription of nilotinib is accompanied by a black box warning on QT prolongation and risk of sudden death.

## 4. Special Populations

### 4.1. Infants

According to a report from the I-CML Ped Study, the percentage of CML in very young (<3 years) children and adolescents is 4.6%. The youngest patient in the registry was 10 months old. The high level of molecular resistance reported in that cohort could be attributed to insufficient doses of imatinib.

### 4.2. Adolescents and Young Adults (AYAs)

AYAs have been reported by MDACC investigators to have inferior rates of response to TKIs as compared to older adults, however, it is not clear whether this is due to inherent differences in TKI sensitivity or to lack of adherence. Compliance is a major issue in adolescent populations and should be monitored systematically.

### 4.3. Vaccination

Imatinib-induced hypogammaglobulinemia without clinical significance has been described in children. Long-term treatment with imatinib may result to memory B-cell reduction, however, safety of live vaccines was documented in children with CML. Vaccination with inactivated vaccines is allowed. This issue is important when the age of diagnosis precedes or coincides with the primary vaccination program. More apprehension exists for the oral polio vaccine, which should be avoided.

As with all TKIs, special consideration must be given to any additional pharmaceutical compound, with a thorough examination of potential drug interactions.

### 4.4. Reproductive Capacity

Hematologists taking care of children and AYAs with CML should provide information about reproductive capacity both at diagnosis and while accompanying their patients through adulthood. Overall, patients and their families should be encouraged to envision a normal family life, albeit under circumstances of strict disease control and pregnancy planning for female patients. Experience with imatinib and, to a lesser extent, with nilotinib and dasatinib constitutes the body of evidence on possible outcomes of the embryo after exposure during pregnancy [4].

Considering female reproductive capacity, imatinib may compromise ovarian function. In addition, imatinib is teratogenic when administered during organogenesis. The majority of reported pregnancies under TKIs have resulted in healthy babies but there was an excess risk of skeletal, gastro-intestinal and kidney malformations after exposure to imatinib during pregnancy, especially during the first trimester. For that reason, specific guidelines have been issued for the management of CML during pregnancy and recommend family planning well before conception, including before in vitro fertilization, with substitution of imatinib for interferon alpha during the peri-conceptional period and the first and second trimesters, along with close monitoring of BCR-ABL1. Dasatinib crosses the placenta at all gestational ages; therefore, it should be proscribed during the entire pregnancy. Imatinib may be detected in breast milk and neonatal blood. Overall, all TKIs are contraindicated during pregnancy. As mentioned below, treatment-free remission offers new perspectives to female CML patients for both pregnancy and breastfeeding.

Considering male reproductive function, no testicular toxicity has been observed in boys or rats. Sperm alterations at diagnosis and during treatment with imatinib have been described therefore, and sperm cryopreservation could be considered at diagnosis for pubertal boys. Fathering under imatinib has not been associated with malformation in progeny; therefore, there is no recommendation against imatinib continuation in men desiring procreation. Projecting onto adulthood, the perspective of treatment-free remission with female patients should also be discussed as an opportunity for straightforward pregnancy planning.

In summary, for children and adolescents, the major goal of ‘targeted therapy’ with TKIs is the malignant fatal disease of CML to be a chronic disease with normal life expectancy, normal growth and normal puberty followed by a normal reproductive capacity.

## Data Availability

Not applicable.
